# Molecular Epidemiology of Travel-Associated and Locally Acquired Dengue Virus Infections in Catalonia, Spain, 2019

**DOI:** 10.3390/v17050621

**Published:** 2025-04-26

**Authors:** Jéssica Navero-Castillejos, Adrián Sánchez-Montalvá, Elena Sulleiro, Aroa Silgado, Tomás Montalvo, Laura Barahona, Núria Busquets, José Muñoz, Daniel Camprubí-Ferrer, Manuel Valdivia, Ana Martínez, Maria Assumpció Bou-Monclús, Itziar Martínez-Calleja, Mireia Jané, Cristina Rius, Hernán Vargas-Leguas, Beatriz Escudero-Pérez, Rosa Albarracín, Alexander Navarro, Mireia Navarro, Josep Barrachina, Miguel J. Martínez

**Affiliations:** 1 Department of Clinical Microbiology, Hospital Clínic de Barcelona, 08036 Barcelona, Spain; jessica.navero@isglobal.org (J.N.-C.); beescudero@clinic.cat (B.E.-P.); ralbarra@clinic.cat (R.A.); anavarr2@clinic.cat (A.N.); minavarr@clinic.cat (M.N.); barrachina@clinic.cat (J.B.); 2 Barcelona Institute for Global Health (ISGlobal), Hospital Clínic de Barcelona, Universitat de Barcelona, 08036 Barcelona, Spain; jose.munoz@isglobal.org (J.M.); daniel.camprubi@isglobal.org (D.C.-F.); 3 Infectious Diseases Department, Vall d’Hebron University Hospital, Universitat Autònoma de Barcelona, PROSICS, 08035 Barcelona, Spain; adrian.sanchez.montalva@gmail.com (A.S.-M.); elena.sulleiro@vallhebron.cat (E.S.); aroa.silgado@vhir.org (A.S.); 4 Urban Pest Surveillance and Control Service, Public Health Agency of Barcelona, 08023 Barcelona, Spain; tmontal@aspb.cat (T.M.); lbarahon@aspb.cat (L.B.); 5 IRTA, Centre de Recerca en Sanitat Animal (CReSA, IRTA-UAB), Universitat Autònoma de Barcelona, 08193 Bellaterra, Spain; nuria.busquets@irta.cat; 6 Public Health Agency of Catalonia, Generalitat of Catalonia, 08005 Barcelona, Spain; manuel.valdivia@gencat.cat (M.V.); a.martinez@gencat.cat (A.M.); assumpcio.bou@gencat.cat (M.A.B.-M.); itziar.martinez@gencat.cat (I.M.-C.); mireia.jane@gencat.cat (M.J.); 7 CIBER Epidemiologia y Salud Pública (CIBERESP), Instituto de Salud Carlos III, 28029 Madrid, Spain; crius@aspb.cat; 8 Epidemiology Service, Public Health Agency of Barcelona, 08023 Barcelona, Spain; havargas@aspb.cat; 9 Centro de Investigación Biomédica en Red de Enfermedades Infecciosas (CIBERINFEC), Instituto de Salud Carlos III, 28029 Madrid, Spain

**Keywords:** dengue, autochthonous transmission, surveillance, *Aedes albopictus*, molecular epidemiology, phylogenetic analysis

## Abstract

Dengue virus (DENV) is the most important arbovirus worldwide. In 2019, a significant increase in dengue cases was reported worldwide, resulting in a peak of imported cases in some European countries such as Spain. We aimed to describe travel-associated and locally acquired DENV strains detected in 2019 in the Catalonia region (northeastern Spain), a hotspot for dengue introduction in Europe. Through sequencing and phylogenetic analysis of the envelope gene, 75 imported viremic cases and two local strains were described. Autochthonous transmission events included an infection of a local mosquito with an imported dengue strain and a locally acquired human dengue infection from a locally infected mosquito. Overall, all four DENV serotypes and up to 10 different genotypes were detected. Phylogenetic analysis revealed transcontinental circulations associated with DENV-1 and DENV-2 and the presence of DENV-4 genotype I in Indonesia, where few cases had been previously described. A molecular study of the autochthonous events determined that local *Ae. albopictus* mosquitoes were infected by an African DENV-1 genotype V strain, while the locally acquired human case was caused by a DENV-3 genotype I of Asian origin. These findings underline the wide variability of imported strains and the high risk of DENV introduction into this territory, emphasizing the importance and usefulness of molecular characterization and phylogenetics for both local and global surveillance of the disease.

## 1. Introduction

Dengue is a viral vector-borne disease endemic in more than 100 countries through tropical and subtropical areas, especially in Southeast Asia and the Americas, among others. It is frequently an asymptomatic infection, and the clinical spectrum of the disease ranges from mild to severe syndrome that can be fatal [1,2]. Dengue is caused by any of the four different serotypes of dengue virus (DENV-1, DENV-2, DENV-3 and DENV-4), each of which is further subdivided into distinct genotypes [3,4]. Although recovery from infection provides long-lasting immunity against that serotype, secondary infection by a different serotype is the major risk factor for developing severe dengue. There are other factors, such as viral genetics and the host immune system, that also influence the disease severity [1,5]. There is no specific antiviral treatment for the infection, and the diagnosis is based on viral detection assays during the acute viremic phase or serological tests [6].

DENV is mainly transmitted through the bite of infected *Aedes* mosquitoes [2]. Although *Ae. aegypti* is the main vector worldwide, the virus can also be transmitted by *Ae. albopictus* [2], an invasive species that spread through the globe during the last decades. Currently, up to 390 million DENV infections may occur annually, and it is estimated that about half of the world’s population is at risk of infection [1]. Because many cases are asymptomatic or misdiagnosed, dengue is considered an under-reported disease [1].

Dengue also has an alarming public health impact in non-endemic countries. The presence of *Ae. albopictus* in some European countries and the increase in imported cases have led to local viral transmission during the last decades [7]. Infected travelers in the acute phase of the illness (when high amounts of virus are present in the bloodstream) can be bitten by local mosquitoes. If these mosquitoes become infected, then they can transmit the disease to other residents and start local dengue transmission. The fact that asymptomatic infections can lead to mosquito infections similar to symptomatic cases represents a challenge for public health surveillance programs [1,8]. In Spain, the first autochthonous DENV transmission event between an infected human and a local mosquito was described in 2015 [9], while the first locally acquired human cases were reported in 2018 [10]. In 2019, of the 5.2 million dengue cases reported worldwide, 4020 were confirmed in Europe, where 12 locally acquired dengue cases occurred, including in Spain [1,11].

Molecular epidemiology is crucial to improving our knowledge of DENV evolution and diversity as well as a useful source of information for the design of public health strategies focused on the prevention and control of travel-associated infections [4]. It is also an essential tool both for the surveillance of imported DENV strains and for the investigation of DENV autochthonous outbreaks or introductions that occurred in non-endemic areas [12,13]. In this study, we describe local transmission events and the molecular epidemiology of acute viremic dengue infections in Catalonia (northeastern Spain), a high-risk area for DENV introduction [14].

## 2. Materials and Methods

### 2.1. Study Design

Dengue cases were detected at participating hospitals in the public health arbovirus surveillance and control program of the Public Health Agency of Catalonia (PHAC) in 2019. Depending on the days between the onset of symptoms and sample collection, molecular and/or serological assays were performed for the diagnosis of dengue, chikungunya and Zika viruses. According to the diagnostic guidelines, dengue cases were classified as confirmed cases when RT-PCR and/or NS1 antigen testing was positive. Cases in which only serological testing was performed with a positive IgM result were classified as probable cases. For this study, only patients with acute DENV infections confirmed by a positive RT-PCR result were included. Demographic and epidemiological data, such as travel date and destination, age and sex, were also reviewed. For a more informative analysis of sources of importation, included DENV cases were classified according to the World Health Organization regions (WHO regions), which group more than 150 countries into 6 different regions to develop preventive strategies for major health emergencies, coordinate prevention programs and design surveillance strategies for risk assessment, all under a single health office.

Following the public health protocol, both suspected and confirmed cases were notified to the PHAC, which, in turn, activated entomological surveillance and vector control measures. Based on the information collected in the epidemiological survey, an entomological survey was carried out in the public space around the place of residence of the case and in the patient’s own home. Subsequently, 10 households near the case were inspected to assess neighborhood activity and breeding sites. Sewers, ornamental fountains and small artificial containers were examined within a radius of 150 m in relation to the case’s place of residence (usual flight rate of *A. albopictus* in the urban environment). Specific treatments to diminish vector abundance were administered, and BG traps were set up to capture adult mosquitoes to be tested for the presence of arboviruses. Details of these procedures have been previously described [9,15].

In our study, information notes with recommendations to prevent the proliferation of larval breeding sites in homes were distributed during inspections of the most vulnerable residential areas near the imported case. These activities are essential to reduce the risk of disease spread, as contact between the vectors and the infected host is likely to occur in the home. Entomological material collected was examined and identified, and females were tested in pools for the presence of the virus by RT-PCR. The surveillance program for imported cases of arbovirosis is integrated into a general procedure for monthly surveillance and treatment of the areas.

The study was conducted according to the guidelines of the Declaration of Helsinki and approved by the Clinical Research Ethics Committee of Hospital Clinic de Barcelona (file HCB/2021/0709). Informed consent for publication was obtained from the locally acquired case patient described in this manuscript.

### 2.2. Viral Genome Detection, Dengue Serotyping and Sequencing

Molecular methods for DENV detection and typing have been detailed previously [16]. Briefly, viral RNA was isolated from 500 µL of serum samples and tested by a commercial assay for DENV (LightMix Modular Dengue, TIB Molbiol, Berlin, Germany). Serotyping of DENV-positive samples was performed using the Centers for Disease Control and Prevention (CDC) DENV 1-4 Real-Time RT-PCR Multiplex Assay or using an in-house generic flavivirus RT-PCR assay [17] followed by Sanger sequencing of the amplicon.

For genotyping and the analysis of the viral strains, we performed nested RT-PCR amplification of the complete virus envelope (E) gene. Different serotype-specific primer sets were used to obtain a genome fragment of approximately 1600 bp. For samples with low viral load, another protocol based on the amplification of the E-non-structural protein 1 (E-NS1) junction was used. This alternative allowed us to obtain a 500 pb genome fragment when amplification of the whole E gene failed. All primers for both amplification and sequencing, as well as the thermal cycler profiles for amplification of the RT-PCR and nested-PCR protocols, were previously described [16]. Amplified PCR products were verified by 1% agarose electrophoresis prior to Sanger sequencing. In order to optimize the assay, a new forward primer was generated for DENV-1 RT-PCR, and, in the case of DENV-2, the RN2C primer was used for RT-PCR and the RRT2 primer for nested PCR (see Supplemental Table S1 for a primer description).

### 2.3. Phylogenetic Analysis

A collection of available sequences representative of all four DENV serotypes and genotypes was extracted from GenBank (https://www.ncbi.nlm.nih.gov/genbank/, accessed on 26 April 2024) to be used as reference sequences for phylogenetic analysis. For each new DENV strain detected in our center, forward and reverse sequences obtained from Sanger sequencing were aligned by the MUSCLE method using MEGAX software (https://www.megasoftware.net/ accessed on 20 January 2025), thus generating the consensus sequence. Then, all consensus sequences were aligned with the corresponding reference sequences according to their serotype using the same MUSCLE tool (https://www.ebi.ac.uk/jdispatcher/msa/muscle?stype=protein accessed on 20 January 2025). Phylogenetic trees were constructed with MEGAX software using the best nucleotide substitution model proposed by the Maximum Likelihood method for a Neighbor-joining tree. The Tamura-Nei (TN93) model and the Kimura-2-parameter (K2) were the best-fit models proposed for the complete E gene and E/NS1 phylogenetic analysis, respectively. In all cases, the analysis was performed with partial gap deletion, applying a Site Coverage Cutoff of 95% in order to maintain the highest reliability of the sequences obtained, reducing the impact of possible errors/gaps intrinsic to the sequencing process itself. Additionally, a bootstrap with 1000 replicates was selected to assess the reliability of the generated phylogenetic trees. For this analysis, newly sequenced DENV strains without travel data were excluded (n = 10).

### 2.4. Data Analysis and Graphs

Excel software version 2024, the Google Drive application and MapChart web (https://www.mapchart.net/world.html), accessed on 10 April 2024, were used for the generation of the tables, graphs and maps included in this manuscript.

## 3. Results

### 3.1. Epidemiology of Imported Cases

**Figure 1:**
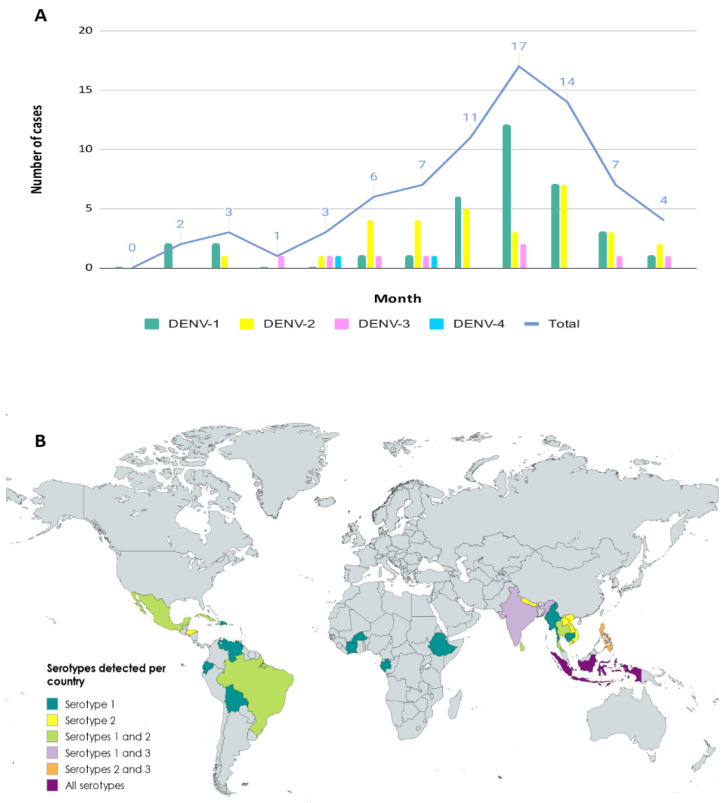
Distribution of RT-PCR-positive dengue serotyped cases detected in Catalonia by month (**A**) and by country’s source of importation (**B**). In this work, no dengue cases were imported from either the Eastern Mediterranean or European regions.

In 2019, 313 suspected cases of acute dengue were tested by molecular methods at our center, of which 75 were confirmed by a positive RT-PCR result. The median age was 37 years (ranging from 75 to 1 years old), and 38.7% were female. Most cases were detected between August and October (56%), and 22.7% of the cases were diagnosed in September. As *Ae. albopictus* mosquito is a species with a marked seasonality modulated according to climatic conditions (mainly temperature and rainfall). Its activity is high in the periods from June to October, with September being a month in which, if the right conditions coincide, a peak activity could be observed in our region (Figure 1A) [18,19].

Accurate travel history was obtained from most (84%) of the patients in order to classify the detected strains according to the WHO regions. According to our results, 46% of imported dengue cases came from the Southeast Asian Region (SEAR), which was the most common area of importation, followed by the Region of the Americas (AMR) (36.5%). Although we had cases from other WHO regions like Africa (AFR) and Western Pacific (WPR) regions, they accounted for less than 7% (6.3% and 4.8%, respectively). Nearly 6% of patients had visited two different WHO regions during their trip (Southeast Asian and Western Pacific regions). No positive cases were detected in travelers returning from either the Eastern Mediterranean Region (EMR) or the European Region (EUR) (Figure 1B).

### 3.2. Serotyping and Genotyping of Imported Cases

Molecular serotyping of the 75 imported DENV strains revealed the presence of the four different viral serotypes, with DENV-1 and DENV-2 being the most prevalent (DENV-1 = 35 cases, DENV-2 = 30 cases, DENV-3 = 8 cases and DENV-4 = 2 cases). DENV-1 was the most frequently observed serotype in travelers returning from AMR (n = 14/23), whereas DENV-2 was the main serotype detected in travelers from SEAR (n = 13/29). However, there was also a high number of DENV-1 cases (n = 12/29) in this region. The two detected DENV-4 cases were imported from SEAR, and DENV acquired in AFR was caused exclusively by DENV-1. As for DENV-3, it was detected in all WHO regions except AMR and AFR (Figure 1B).

Of the 75 imported dengue cases, 68 could be sequenced for genotyping and phylogenetic analysis (34 DENV-1, 26 DENV-2, 6 DENV-3 and 2 DENV-4). Non-genotyped cases showed a very low viral load, insufficient for amplification and sequencing. Up to 10 different genotypes were identified, with genotype V of DENV-1 (n = 20, 29.4%) being the most frequently detected, followed by DENV-1 genotype I (n = 13, 19%) and DENV-2 cosmopolitan genotype (n = 12, 17.6%). For DENV-3 and DENV-4, only two different genotypes were recognized (genotypes I and III and I and II, respectively). More variability was found in DENV-1, for which three different genotypes were described (I, IV and V). The same was observed with DENV-2, in which American–Asian, Asian I and Cosmopolitan genotypes were reported. The number of cases per genotype for each DENV serotype is shown in Table 1. The sequences obtained from DENV imported strains can be found in the GenBank database with the accession numbers PP434937−PP434986 and PP897390−PP897397 and a description of detected strains is available in Supplemental Table S2.

### 3.3. Phylogenetic Analysis of DENV Imported Strains

**Figure 2:**
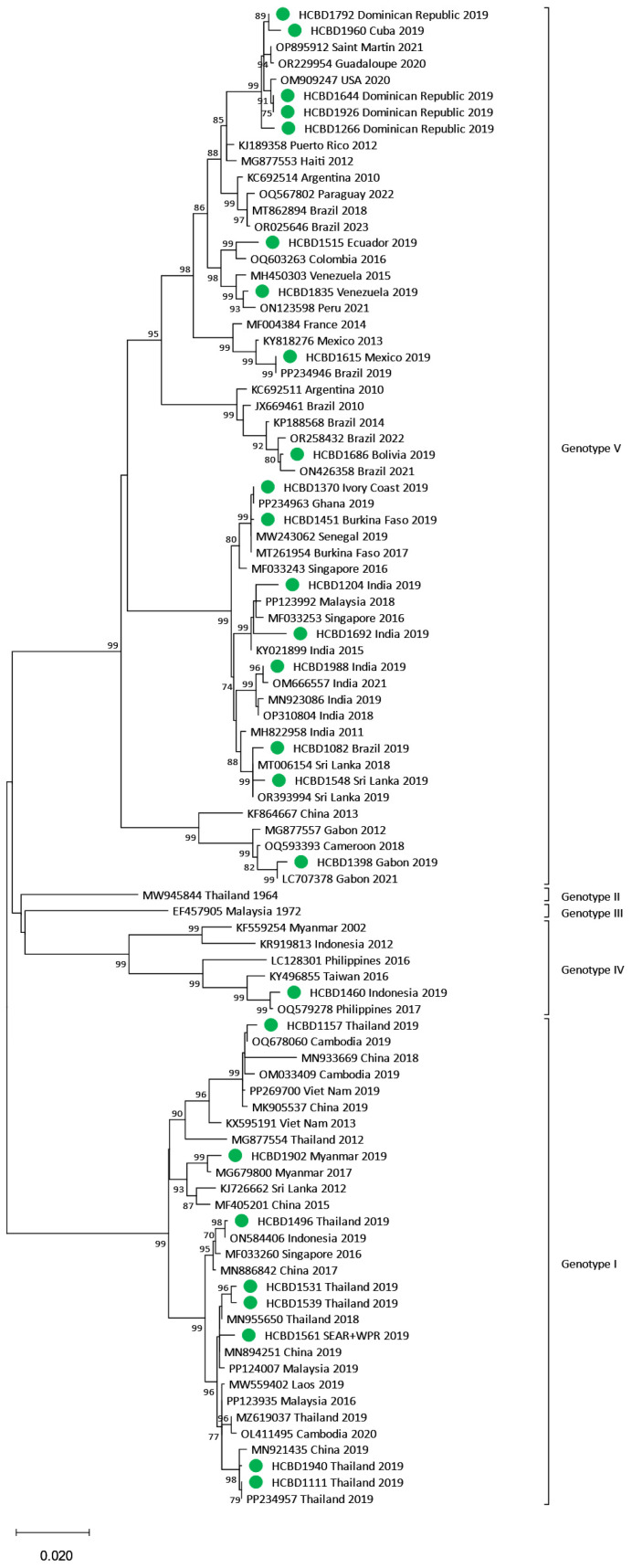
Phylogenetic tree of DENV-1 imported strains based on the E gene. The analysis was performed using a Maximum Likelihood method (TN93 + G) with a bootstrap of 1000 replicates. Sequences obtained in this work are marked with green dots. Reference sequences are described with GenBank accession number, place and year of isolation and the scale bar indicates substitutions per site.

**Figure 3:**
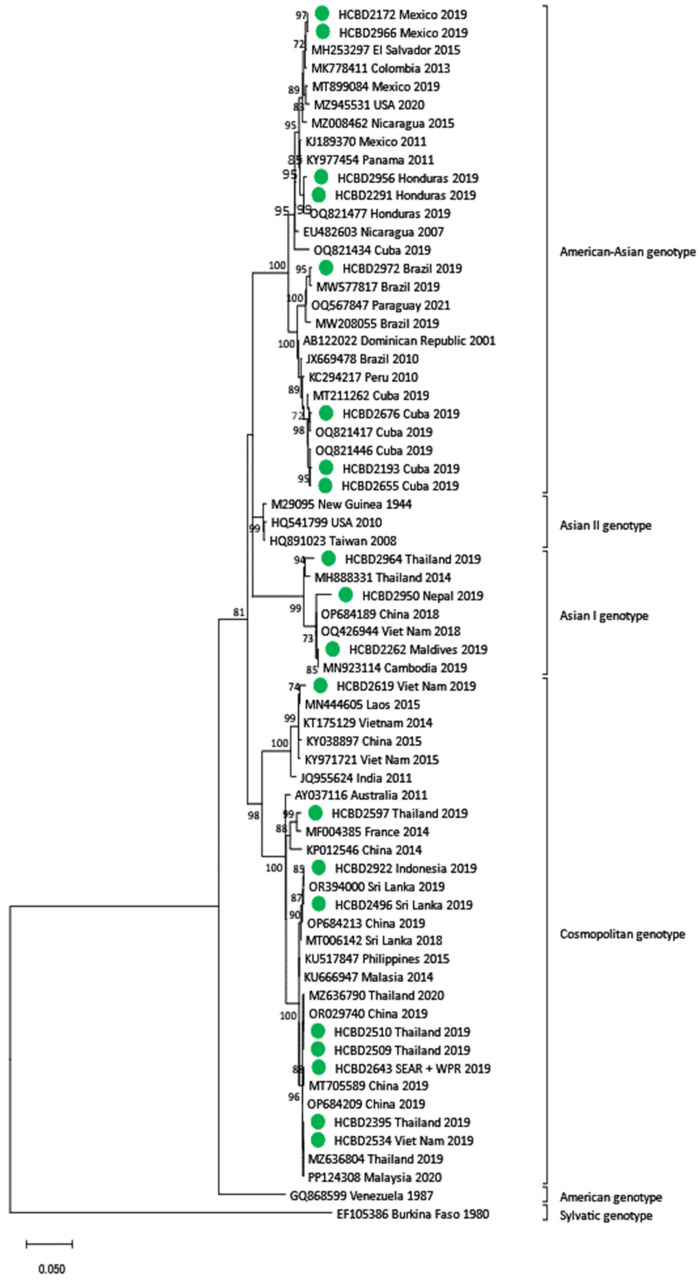
Phylogenetic tree of DENV-2 imported strains based on the E gene. The analysis was performed using a Maximum Likelihood method (TN93 + G + I) with a bootstrap of 1000 replicates. Sequences obtained in this work are marked with green dots. Reference sequences are described with GenBank accession number, place and year of isolation and the scale bar indicates substitutions per site.

**Figure 4:**
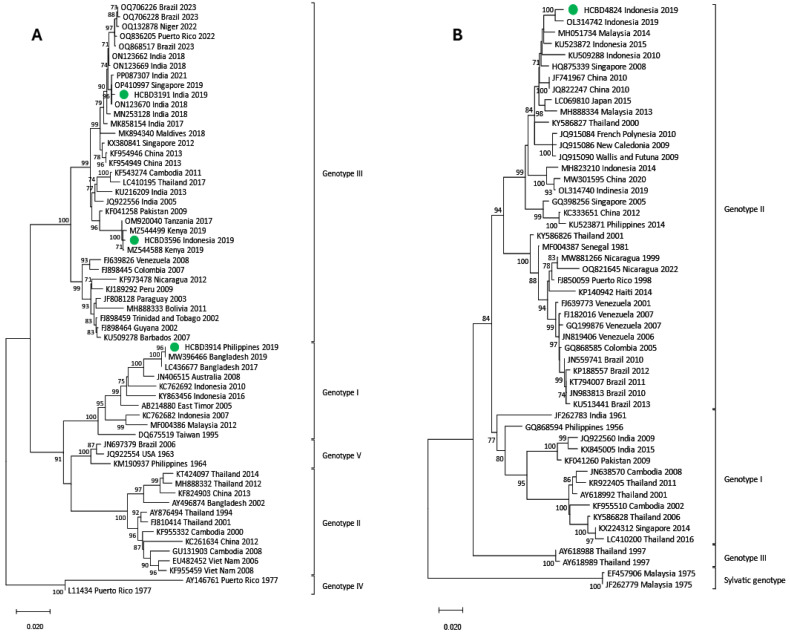
Phylogenetic tree of imported strains based on the E gene. (**A**) Phylogenetic tree of DENV-3 imported strains obtained through the TN93 + G model. (**B**) Phylogenetic tree of DENV-4 imported strains obtained through the TN93 + G + I model. Green dots correspond to samples detected in our center. Reference sequences are described with GenBank accession number, place and year of isolation and the scale bar indicates substitutions per site. The analysis was performed using a bootstrap of 1000 replicates.

**Figure 5:**
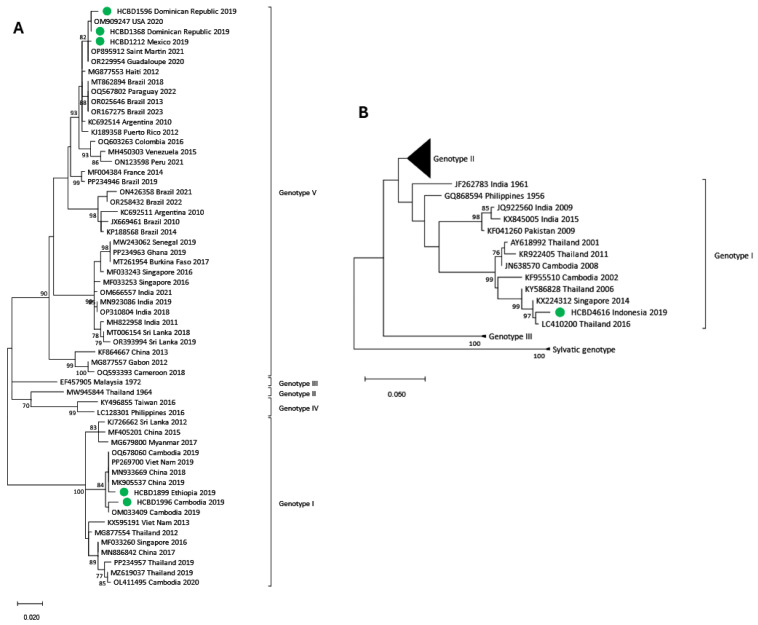
DENV phylogenetic trees based on the E-NS1 junction. (**A**) DENV-1 phylogenetic tree obtained through the K2 + I model. (**B**) DENV-4 phylogenetic tree obtained through the K2 + G model. Green dots correspond to samples detected in our center. Reference sequences only include the E-NS1 junction region targeted by our amplification protocol for low viral load samples. Those without the E-NS1 junction region were excluded in order to optimize the analysis. The analysis was performed using a bootstrap of 1000 replicates.

Phylogenetic trees for each DENV serotype were constructed with the strains sequenced and reference strains selected from the GenBank database. Two phylogenetic trees were performed for each serotype. One of them included strains in which the complete E gene was obtained (n = 50) (Figure 2, Figure 3 and Figure 4), and the other contained strains with low viral load in which the E/NS1 junction could only be sequenced (n = 8) (Figure 5; see Supplemental Figure S1 for DENV-2 and DENV-3 E/NS-1 phylogenetic trees).

All DENV-1 strains imported from American countries clustered within genotype V, while the ones imported from SEAR showed a high variability, clustering into genotypes I, IV and V. Of the four strains from the African region, three were also grouped into genotype V, and the one imported from Ethiopia belonged to genotype I. For DENV-2, the cosmopolitan genotype was the most frequently detected and included strains from SEAR and WPR. The Asian I genotype was found in three samples from Nepal, the Maldives and Thailand and in the Americas, all DENV-2 strains belonged to the American–Asian genotype. DENV-3 acquired in SEAR and WPR belonged to genotypes III and I, respectively. Only two strains of DENV-4 were detected in 2019. Although both came from the SEAR, one belonged to genotype II and the other to genotype I.

### 3.4. Local Transmission Events

#### 3.4.1. Human-to-Mosquito Transmission

**Figure 6:**
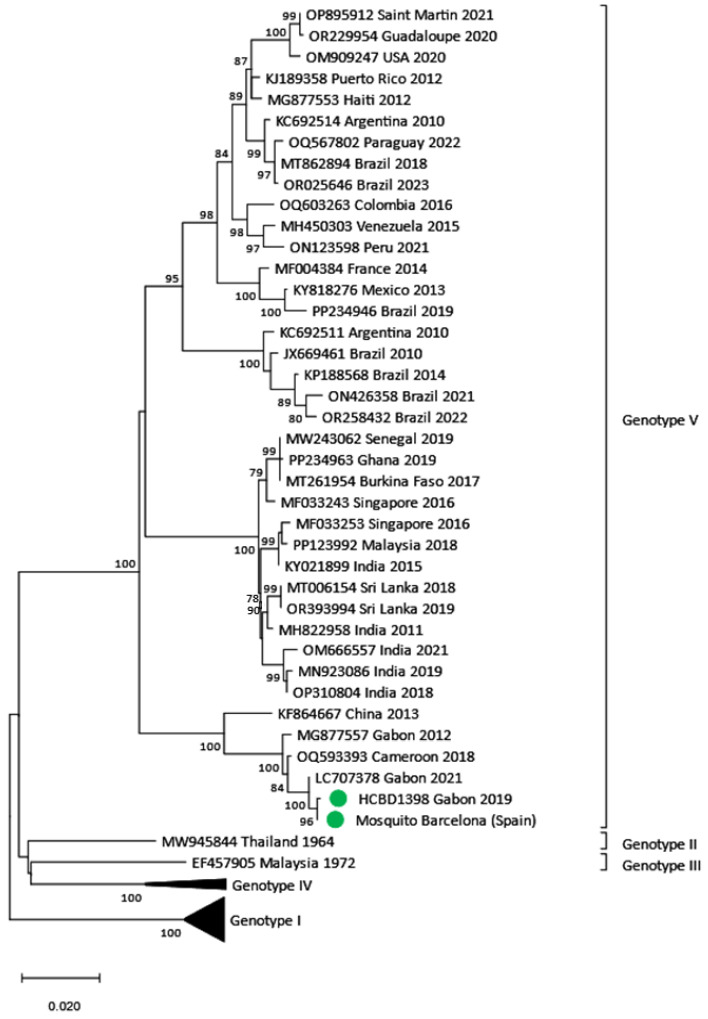
Phylogenetic tree of the DENV-1 autochthonous transmission event based on the E gene. Phylogenetic tree of autochthonous DENV-1 strain obtained from a local mosquito pool (Mosquito Spain sample) and the imported strain from Gabon (HCBD1398 sample). Green dots correspond to samples detected in our center. Reference sequences are noted with the GenBank accession number, place and year of isolation and the scale bar indicates substitutions per site. The analysis was performed using a Maximum Likelihood method (TN93 + G) with a bootstrap of 1000 replicates. The sequence obtained from the DENV mosquito strain can be found in the GenBank database with the accession number PP897402.

Following the entomological surveillance protocol, inspections and capture of female *Ae. albopictus* mosquitoes were to be conducted around the viremic DENV case during the 12 days following symptom onset (22 August). In this case, entomological surveillance started on the same day as case confirmation (29 August), four days after the patient returned from Gabon and seven days after symptom onset. However, monitoring was extended for approximately 20 days after diagnosis due to the detection of vector activity in the area. In addition to the one carried out at the residence of the imported case, 10 other entomological inspections were performed in households in the area. At the case residence, two BG traps were placed in the garden and checked every 24 h since 30 August. The inspection and surveillance effort was intensified when the positive pool was notified (5 September), placing five BG traps in four houses close to the case and included in the radius of 150 m. Mosquitoes were only captured at the residence of the DENV-positive patient. Of the nine mosquito pools collected, comprising a total of 77 *Ae. albopictus*, the positive pool contained 13 individuals and was collected on the fourth day of trapping. Characterization and phylogenetic analysis of the patient’s strain and the strain detected in the mosquito pool showed a DENV-1 genotype V. Comparison of the complete E gene confirmed the identity of both sequences, which were closely related to other strains described in Gabon, Cameroon and China between 2012 and 2021 (Figure 6).

#### 3.4.2. Locally Acquired Dengue Case

**Figure 7:**
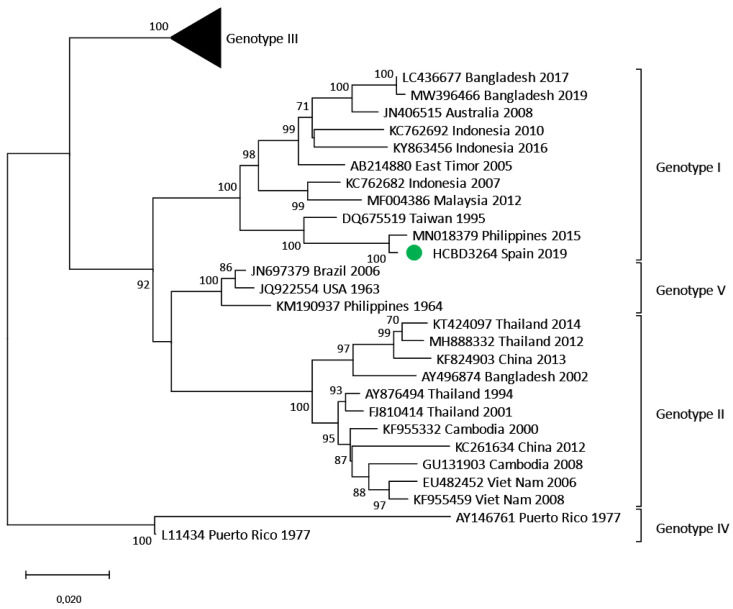
Phylogenetic tree of the locally acquired DENV-3 strain based on the complete E gene. Reference sequences are noted with the GenBank accession number, place and year of isolation and the scale bar indicates substitutions per site. The green dot corresponds to the autochthonous strain detected in our center, and the analysis was performed using a Maximum Likelihood method (TN93 + G) with a bootstrap of 1000 replicates. The sequence obtained from the DENV mosquito strain can be found in the GenBank database with the accession number PP897403.

In September 2019, a young woman without a travel history abroad in the previous two years sought medical attention due to febrile syndrome. Urine and blood tests were performed, showing leukocytes in urine sediment and leukopenia, respectively. Blood culture and renal echography were normal, and the patient was discharged with third-generation cephalosporin treatment to cover a possible urinary infection. Five days later, she presented with a pruritic macular rash on the thorax and abdomen within 48 h of evolution. Blood tests showed leukopenia, thrombocytopenia and hypertransaminasemia. The antibiotic treatment was discontinued, and antihistamines and corticosteroids were prescribed due to the orientation towards an allergic rash. She was directed to the tropical diseases unit, where a dengue virus infection was suspected. Dengue serological tests were carried out, showing IgM and IgG positive results. The viral RNA was also detected through molecular methods (RT-PCR). Eight days after the first medical visit, the patient was afebrile, but the rash and asthenia persisted. The patient was reinterrogated and reported having been bitten by mosquitoes in the metropolitan area of Barcelona during the days before the onset of symptoms. As the patient had no recent history of traveling, a locally acquired dengue case was considered, and retrospective acute samples as well as follow-up samples were obtained. Confirmation of the diagnosis was obtained by detection of DENV RNA in the stored samples from the first days of illness in which IgM and IgG serological tests were negative. Seroconversion of IgG was also detected in the follow-up samples. This represented the second case of locally transmitted dengue infection in the region and the seventh in Spain. DENV-3 genotype I was identified, and no analogous DENV-3 strain was detected in this study. No epidemiologically linked cases were detected that could represent the source of the transmission. Within genotype I, the autochthonous strain clustered with other DENV-3 Asian strains (Figure 7).

## 4. Discussion

Dengue is a growing public health threat in both endemic and non-endemic areas [1]. The wide distribution of competent vectors, the effect of climate change and the international mobility of people are some of the main factors that contribute to the spread of the disease. In Europe, thousands of travelers infected with DENV arrive from endemic areas each year, and many of them are living in areas colonized by the *Ae. albopictus* vector. This facilitates the risk of local transmission in European countries [20]. This situation is favored by high rates of asymptomatic infection and low clinical suspicion, which hampers the diagnosis of an important number of cases and hinders the effectiveness of prevention and control measures [8]. In this scenario, strategies that improve case detection, such as training healthcare workers and the use of rapid diagnostic tests, become essential for the early detection of DENV cases and the on-time implementation of control measures. However, given the challenges in applying these measures, surveillance and characterization of DENV imported strains by international travelers has become an important tool for global dengue surveillance but also for assessing the risk of introduction and transmission in non-endemic countries. In this line, some studies have described a wide variety of travel-related imported DENV strains, including all four serotypes and multiple genotypes [12,20]. Accordingly, our study characterized 75 DENV strains imported from 23 different countries in America, Africa and Asia, detecting all four DENV serotypes and 10 different genotypes. These results underline dengue viral diversity and reflect global dengue circulation in the endemic areas visited by travelers. In addition, we revealed that two autochthonous transmission events were caused by different strains of DENV-1 and DENV-3, highlighting the risk of introduction associated with the importation of multiple variants of the virus in our territory.

Phylogenetic analysis of imported strains can provide relevant epidemiological information about DENV global circulation. Our results showed a higher prevalence of DENV-1 and DENV-2 serotypes, particularly the most widespread genotypes (I and V of DENV-1 and American–Asian and Cosmopolitan of DENV-2). Co-circulation of different serotypes in the same country was previously reported, especially in parts of the Americas and Asia [21]. Our study also revealed this phenomenon by describing cases of all four serotypes imported from Indonesia. Even at the genotype level, co-circulation of DENV-4 genotypes I and II was also observed in that country. Although DENV-4 is less frequently detected globally [21,22], it has caused significant outbreaks in the Americas [23] and Asia [24]. In Indonesia, DENV-4 outbreaks are mainly caused by genotype II, such as the outbreak in Java in 2019 [25], and only a few strains belonging to genotype I are detected [22]. Our results demonstrated that our genotype II imported strain was similar to those reported in the Java Island outbreak. In contrast, the genotype I of the imported strain was derived from those reported in Thailand in 2016, which is in agreement with previous findings [22].

The high number of imported strains, as well as the high diversity between sequences, allowed us to identify the dispersal dynamics of the virus between different countries. The high similarity between DENV-2 cosmopolitan genotype strains imported from Viet Nam and Thailand and others circulating in the area between 2019 and 2020 [26] suggested a sustained circulation of this strain in the area. Furthermore, the spread of a DENV-2 strain responsible for the DENV outbreak in Sri Lanka in 2019 [27], which was detected in a traveler from Indonesia, could also be described. It is important to consider that a new introduction could result in the emergence of a new lineage, as shown in Paraguay in 2021 [28].

In addition to the circulation of the same DENV strain between neighboring countries, our work also confirmed the transcontinental spread of DENV-3 and DENV-1. Between 2017 and 2019, DENV-3 genotype III outbreaks occurred in Kenya [29] and Tanzania [30]. In both cases, the genealogical ancestry was attributed to strains circulating in Pakistan in recent decades. Our strain imported from Indonesia was nested in this cluster, suggesting that the strain responsible for the Tanzanian outbreaks spread to Kenya and was also circulating in Southeast Asia. The introduction of the DENV-1 genotype V in the Americas from a probably Indian strain has been previously reported [31]. A similar situation could occur in 2019, explaining the similarity between the strain detected in a traveler from Brazil and those described during 2018–2019 in Sri Lanka. In Ethiopia, dengue outbreaks have been increasing in recent years. Although serotypes 1, 2 and 3 are known to circulate [32,33], there is little sequence information on these viruses. Our results show a strong similarity between the DENV-1 genotype I strain imported from Ethiopia and Asian strains, denoting a possible new introduction event. Taken together, molecular characterization of dengue in travelers reflected the global circulation of different strains.

Dengue acquired in Africa by European travelers is seldom observed compared to cases following trips to Asia and America. The DENV strains imported from Africa in our study included, in addition to the Ethiopian strain, three DENV-1 genotype V strains. Two of them, imported from Burkina Faso and Ivory Coast, were grouped in the African monophyletic group within the Asian lineage of genotype V, as other imported strains into other countries [34]. These strains, which have been detected in West African countries relatively recently, appear to have been introduced from Asia [34,35]. The third African DENV-1 genotype V case, returning from Gabon, led to the detection of infected mosquitoes and clustered with different genotype V strains circulating in West Africa and Asia, although it was similar to those reported in Cameroon in 2018 [36] and in Gabon in 2012 and 2021 [37]. This was the second event of local transmission described in our region between a dengue-positive traveler and a local *Ae. albopictus* mosquito population. Detection of infected vectors is rarely reported in Europe and may underline the timely implementation of entomological control measures following the detection of a dengue case in our surveillance program. Capturing infected female *Ae. albopictus* for testing may contribute to a better risk assessment and limit the spread of the disease.

Since the first locally acquired cases in Croatia in 2010, locally transmitted dengue cases have been increasingly recorded in Europe [7]. In 2018, the first six locally acquired dengue cases occurred in Spain, five reported in Murcia and one in Catalonia. Although the detected strains were different, all were DENV-1 viruses likely imported from Asia [10]. The case presented in this study and a sexually transmitted case in Madrid were the sole locally acquired cases detected in 2019 in Spain [11]. In non-endemic regions, the primary index case of autochthonous events is defined as the infected traveler that led to mosquito infection and subsequent transmission. So far, in all locally acquired cases that occurred in Spain, as well as in most of those detected in Europe, the primary index case has not been identified. This can be explained, among other factors, because undetected asymptomatic infections seem to be equally infectious for the vectors as symptomatic cases [8] and because of the unspecific clinical presentation of dengue [2], which can lead to misdiagnosis with other tropical diseases. The last applies to the challenges in the detection of locally transmitted cases. The locally acquired case presented here was consulted several times until dengue was suspected. The same was observed in the first locally acquired case in our region in 2018 [16] and highlights the urgent need to increase clinical awareness and laboratory preparedness for arboviral surveillance in *Ae. albopictus* colonized areas.

## 5. Conclusions

In conclusion, our study analyzes the impact of a high dengue epidemic year in a non-endemic area colonized by *Ae. albopictus* where monitoring for local transmission is carried out. Our results highlight the utility of vectorial and molecular investigations for dengue to accurately describe autochthonous transmission events. As dengue is globally increasing, reinforcement of clinical and virological surveillance is needed through a One Health approach that incorporates environmental, animal and human health.

## Figures and Tables

**Table 1:** Number of characterized DENV cases per genotype for each serotype.

DENV Serotype	DENV Genotype	Number of Cases
DENV-1 (n = 34)	I	13
	IV	1
	V	20
DENV-2 (n = 26)	American–Asian	11
	Asian I	3
	Cosmopolitan	12
DENV-3 (n = 6)	I	3
	III	3
DENV-4 (n = 2)	I	1
	II	1

## Data Availability

All relevant data are within the paper. All DENV sequences obtained in this study can be accessed at GenBank Table S2 (supplementary material).

## Supplementary Materials

The following supporting information can be downloaded at https://www.mdpi.com/article/10.3390/v17050621/s1, Table S1: Primers used for E and E/NS1 junction amplification; Table S2: Characteristics of strains described in this work; Figure S1: DENV2 and DENV-3 phylogenetic trees based on the E-NS1 junction.
