# Supplementary material for: Molecular Epidemiology of Travel-Associated and Locally Acquired Dengue Virus Infections in Catalonia, Spain, 2019
Source: Viruses. 2025 Apr 26;17(5):621. doi: 10.3390/v17050621 (PMC12115671; doi:10.3390/v17050621)

Supplementary materials

Supplemental Tables

Table S1. Primers used for E and E/NS1 junction amplification. DENV-2 rimers whose order was modified with respect to the reference protocol are marked in bold. The * marks the new DENV-1 primer used.

				Primer	Sequence (5'-3')	Localization
E	DENV-1	RT-PCR	F	RFT1*	GAAACRTGGATGTCHTCYGA	758-778
			R	EGENE-R	TCCTCCCATGCCTTCCCRATGG	2553-2574
		Nested-PCR	F	F Nested	ATAGGAACATCCATYACYCAG	866-887
			R	EGENE/NS-RR	TGRAAYTTRTAYTGYTCTGTCC	2502-2523
	DENV-2	RT-PCR	F	EGENE2-S	CTGAAACATGGATGTCATCAGAAGG	758-782
			R	**RNC 2**	**GCYGAWGCYARYTTTGRRGGRG**	**2534-2555**
		Nested-PCR	F	F Nested	ATGGCRGCDATYYTGGCDYAY	844-865
			R	**RRT 2**	**GCYGARGCYARYTTTGARGGRG**	**2533-2555**
	DENV-3	RT-PCR	F	EGENE3-S	CTCAAACCTGGATGTCGGCTGARGG	756-780
			R	RRT 3	ATYCCRCAVACTCCATTYTYCC	2561-2583
		Nested-PCR	F	F NESTED	ATGYTGGTCACYCCATCCATG	911-932
			R	R NESTED	TTGTAYTGYTCTGTCCARGTRTG	2511-2534
	DENV-4	RT-PCR	F	EGENE4-S	CTGAGACATGGATGTCATCGGAAGG	760-784
			R	RRT 4	CACAGACCCCHTCTTTGTGRGC	2567-2589
		Nested-PCR	F	F NESTED	TACTCAGRAABCCAGGATTYGC	869-890
			R	R NESTED	YTCCATGACACYRCACAACCC	2478-2470
E/NS1	DENV-1	RT-PCR	F	FRTC 1	TGSYTGAGACYCARCAYGGNAC	1869-1890
			R	RRTC 1	YTCRTTTGATATYTGYYTCCAC	2620-2641
		Nested-PCR	F	FNC 1	GRAAATGTTYGARGCHACYGCCC	2130-2153
			R	RNC 1	TCYTCCCAYGCYYTYCCRATGG	2553-2574
	DENV-2	RT-PCR	F	FRTC 2	TAGCWRRRACRCARCATGGAAC	1871-1889
			R	RRTC 2	CAGTTCYGGWGYTATYTGYYTCCAC	2622-2646
		Nested-PCR	F	FNC 2	CARTYARYATAGAAGCAGARCC	2027-2048
			R	RNC 2	GCYGAWGCYARYTTTGRRGGRG	2534-2555

DENV-3	RT-PCR	F	FRTC 3	TYTCHGARACRCARCAYGGRAC	1863-1884
		R	RRTC 3	BARYTCATTRGCTAYTTGCTTCCAY	2614-2638
	Nested-PCR	F	FNC 3	GRRAARATGTTYGAGRCSMCYG	2125-2146
		R	RNC 3	ATYCCRCAVACTCCATTYTYCC	2562-2583
DENV-4	RT-PCR	F	FRTC 4	TGGCAGAAACACARCATGGRAC	1873-1894
		R	RRTC 4	YARYTCRTTRGTTATTTGYTTCCAC	2624-2648
	Nested-PCR	F	FNC4	ATYGGYAAGATGTTYGAGTCY	2130-2150
		R	RNC 4	CACAGACCCCHTCTTTGTGRGC	2568-2589

* marks the new DENV-1 primer used.

Table S2. Characteristics of strains described in this work.

Strain	Serotype	Genotype	Country	Sequence	GenBank ID
HCBD1531	1	I	Thailand	E	PP434948
HCBD1539	1	I	Thailand	E	PP434949
HCBD1899	1	I	Ethiopia	E-NS1	PP897393
HCBD1996	1	I	Cambodia	E-NS1	PP897394
HCBD1157	1	I	Thailand	E	PP434939
HCBD1902	1	I	Myanmar	E	PP434958
HCBD1111	1	I	Thailand	E	PP434938
HCBD1940	1	I	Thailand	E	PP434960
HCBD1561	1	I	SEAR+WPR	E	PP434951
HCBD1496	1	I	Thailand	E	PP434946
HCBD1460	1	IV	Indonesia	E	PP434945
HCBD1835	1	V	Venezuela	E	PP434957
HCBD1596	1	V	Dominican Republic	E-NS1	PP897392
HCBD1988	1	V	India	E	PP434962
HCBD1792	1	V	Dominican Republic	E	PP434956
HCBD1615	1	V	AMR	E	PP434952
HCBD1548	1	V	Sri Lanka	E	PP434950
HCBD1926	1	V	Dominican Republic	E	PP434959
HCBD1368	1	V	Dominican Republic	E-NS1	PP897391
HCBD1204	1	V	India	E	PP434940
HCBD1266	1	V	Dominican Republic	E	PP434941
HCBD1692	1	V	India	E	PP434955
HCBD1212	1	V	Mexico	E-NS1	PP897390
HCBD1644	1	V	Dominican Republic	E	PP434953

HCBD1686	1	V	Bolivia	E	PP434954
HCBD1451	1	V	Burkina Faso	E	PP434944
HCBD1515	1	V	Ecuador	E	PP434947
HCBD1398	1	V	Gabon	E	PP434943
Mosquito Pool Barcelona 2019	1	V	Spain	E	PP897402
HCBD1370	1	V	Ivory Coast	E	PP434942
HCBD1082	1	V	Brazil	E	PP434937
HCBD1960	1	V	Cuba	E	PP434961
HCBD2972	2	American-Asian	Brazil	E	PP434982
HCBD2291	2	American-Asian	Honduras	E	PP434966
HCBD2676	2	American-Asian	Cuba	E	PP434976
HCBD2655	2	American-Asian	Cuba	E	PP434975
HCBD2956	2	American-Asian	Honduras	E	PP434979
HCBD2190	2	American-Asian	Mexico	E-NS1	PP897395
HCBD2172	2	American-Asian	Mexico	E	PP434963
HCBD2193	2	American-Asian	Cuba	E	PP434964
HCBD2966	2	American-Asian	Mexico	E	PP434981
HCBD2395	2	Cosmopolitan	Thailand	E	PP434967
HCBD2496	2	Cosmopolitan	Sri Lanka	E	PP434968
HCBD2534	2	Cosmopolitan	Viet Nam	E	PP434971
HCBD2643	2	Cosmopolitan	SEAR+WPR	E	PP434974
HCBD2619	2	Cosmopolitan	SEAR	E	PP434973
HCBD2509	2	Cosmopolitan	Thailand	E	PP434969
HCBD2510	2	Cosmopolitan	Thailand	E	PP434970
HCBD2597	2	Cosmopolitan	Thailand	E	PP434972
HCBD2922	2	Cosmopolitan	Indonesia	E	PP434977
HCBD2950	2	Asian I	Nepal	E	PP434978
HCBD2262	2	Asian I	SEAR	E	PP434965
HCBD2964	2	Asian I	SEAR	E	PP434980
HCBD3914	3	I	Philippines	E	PP434985
HCBD3517	3	I	Spain	E	PP897403
HCBD3908	3	III	SEAR+WPR	E-NS1	PP897397
HCBD3191	3	III	India	E	PP434983
HCBD3596	3	III	Indonesia	E	PP434984
HCBD4616	4	I	Indonesia	E-NS1	PP897396
HCBD4824	4	II	Indonesia	E	PP434986

Supplemental Figures

Figure S1. DENV phylogenetic trees based on the E-NS1 junction: (**A**) DENV-2 phylogenetic tree obtained though K2+I model. (**B**) DENV-3 phylogenetic tree obtained though K2+I model. Green dots correspond to samples detected in our center. Reference sequences without E-NS1 junction region were excluded in order to optimize the analysis. A bootstrap of 1000 replicates was used.

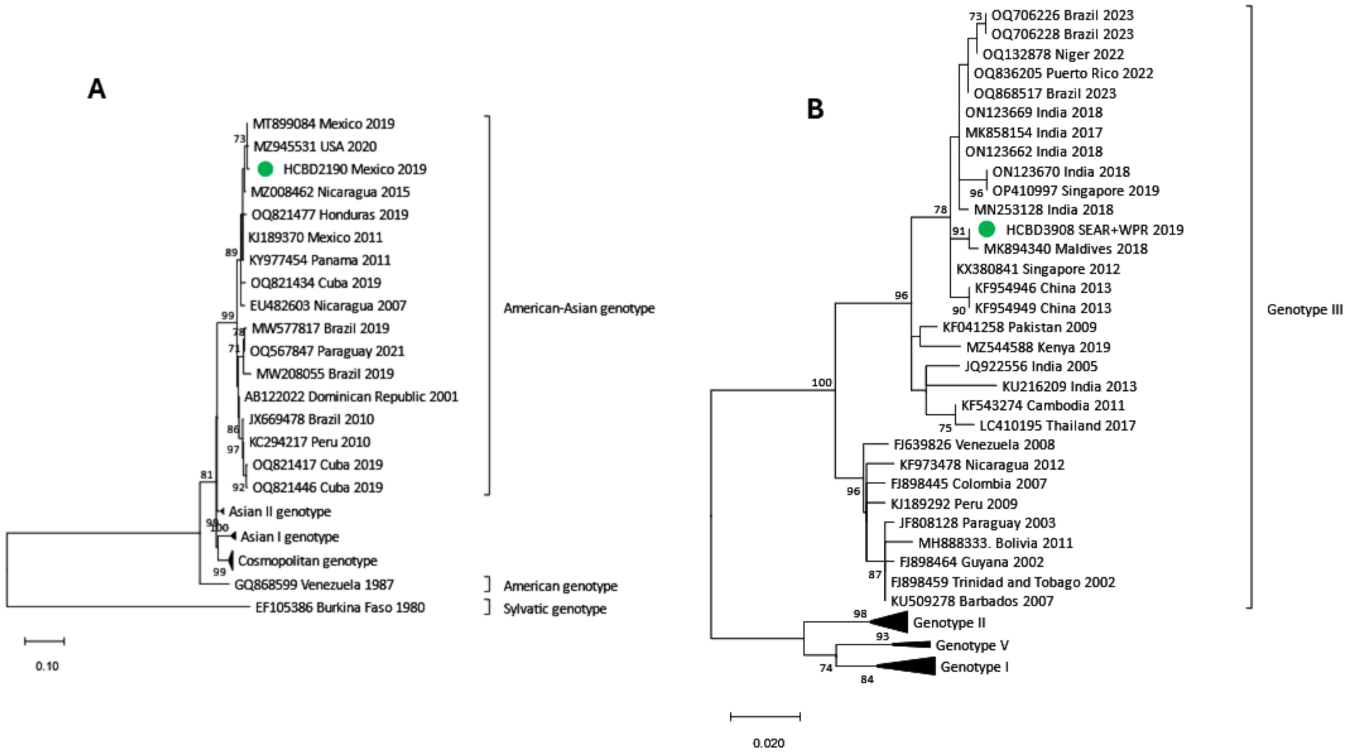

Supplement: Supplementary file 1 [file viruses-17-00621-s001.zip › viruses-3560675-supplementary.pdf]
